# Stereotactic Body Radiation Therapy versus Surgical Resection for Stage I/II Hepatocellular Carcinoma

**DOI:** 10.3390/cancers15082330

**Published:** 2023-04-17

**Authors:** Emrullah Birgin, Svetlana Hetjens, Moses Tam, Camilo Correa-Gallego, Nuh N. Rahbari

**Affiliations:** 1 Department of Surgery, Universitätsmedizin Mannheim, Medical Faculty Mannheim, Heidelberg University, 68167 Mannheim, Germany; 2 Department of Medical Statistics and Biomathematics, Medical Faculty Mannheim, Heidelberg University, 68167 Mannheim, Germany; 3 Department of Radiation Oncology, NYU Grossman School of Medicine, New York, NY 10016, USA; 4 Department of Surgery, NYU Grossman School of Medicine, New York, NY 10016, USA

**Keywords:** SBRT 1, liver resection 2, multimodal 3, ablative 4, HCC 5

## Abstract

**Simple Summary:**

The treatment of localized liver cancer remains a persistent clinical challenge. This study compared surgical resection to stereotactic body radiation therapy and demonstrated a prolonged overall survival after surgery. The use of SBRT needs to be evaluated in prospective trials and should be limited to patients who are not surgical candidates.

**Abstract:**

SBRT is an emerging locoregional treatment modality for hepatocellular carcinoma (HCC). Although local tumor control rates seem encouraging, large-scale survival data comparing SBRT to surgical resection are lacking. We identified patients with stage I/II HCC from the National Cancer Database amenable for potential surgical resection. Patients undergoing hepatectomy were matched by propensity score (1:2) with patients who underwent SBRT as primary treatment. A total of 3787 (91%) and 366 (9%) patients underwent surgical resection or SBRT between 2004 and 2015, respectively. After propensity matching, the 5-year overall survival was 24% (95% CI 19–30%) in the SBRT group versus 48% (95% CI 43–53%) in the surgery group (*p* < 0.001). The association of surgery with overall survival was consistent in all subgroups. In patients treated with SBRT, a biologic effective dose (BED) of ≥100 Gy (31%, 95% CI 22%–40%) compared with BED < 100 Gy (13%, 95% CI 8–22%) was associated with a higher 5-year overall survival rate (hazard ratio of mortality of 0.58, 95% CI 0.43–0.77; *p* < 0.001). Surgical resection may be associated with prolonged overall survival compared with SBRT in patients with stage I/II HCC.

## 1. Introduction

Hepatocellular carcinoma (HCC) accounts for 90% of primary liver malignancies, which is the fourth leading and fastest-growing cause of cancer-related deaths worldwide [1]. Over the last three decades, the global HCC incidence and mortality rates have more than doubled [2,3]. HCC management is highly complex and depends on tumor burden and patients’ risk factors. According to the latest AASLD (American Association for the Study of Liver Diseases), EASL (European Association of the Study of the Liver), Japan Society of Hepatology (JSH), and NCCN (National Comprehensive Cancer Network) guidelines, surgical resection remains the first-line treatment of localized HCC [4,5,6,7]. Other locoregional treatments such as ablative therapies and stereotactic body radiation therapy (SBRT) have traditionally been offered to patients who were no candidates for surgery. 

Historically, the use of radiation therapy has been omitted in the management of HCC due to concerns of radiation-induced liver toxicity [8,9]. However, advances in radiation techniques have enabled the targeted delivery of high-radiation doses by SBRT to liver lesions [10]. Recently, the use of SBRT has gained increasing popularity in the treatment of localized HCC due to reports of high local tumor control rates of up 91% within three years [11,12]. Moreover, three retrospective cohort studies compared SBRT as a noninvasive alternative to surgical resection in early-stage HCC, with promising results [13,14,15]. However, these analyses were limited to single institutions and relatively small cohorts and lacked long-term survival data. While there is some evidence for a survival benefit from randomized trials comparing surgery to radiofrequency and microwave ablation, high-level evidence or large-scale data comparisons to other locoregional treatments including SBRT are lacking [16,17]. In addition, a recent update of the Barcelona Clinic Liver Cancer (BCLC) algorithm model endorses a shift towards personalized treatment strategies [18]. To evaluate SBRT as an alternative and additional treatment option to surgical resection in patients with stage I/II HCC, we analyzed the National Cancer Database (NCDB) with propensity score matching.

## 2. Materials and Methods

Ethical review and approval were waived for this study owing to the use of de-identified data from a public database. This project was conducted in accordance with the Declarations of both Helsinki and Istanbul. The NCDB is a prospectively maintained cancer registry gathering data from more than 1500 accredited hospitals in the United States [19]. 

**Figure 1:**
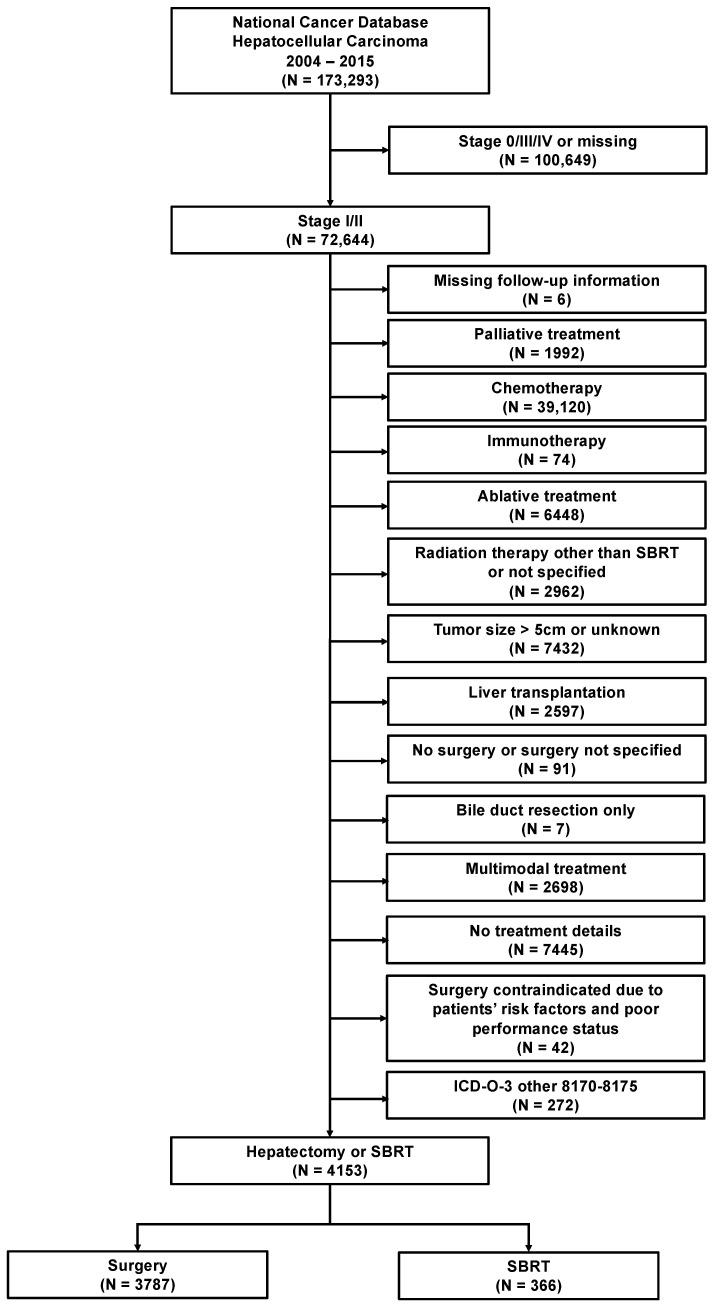
Study flow diagram.

Using the liver participant user file (PUF), we identified a total of 72,644 patients with HCC clinical stage I/II from a population of 173,293 patients with primary HCC diagnosis between 2004 and 2015. The study flow diagram is displayed in Figure 1 in line with the STROBE (Strengthening the Reporting of Observational Studies in Epidemiology) guideline [20]. Patients with clinical stage I and stage II based on T, N, and M elements, as well as tumor size < 5 cm, as defined by the American Joint Committee on Cancer (AJCC) Staging Manual, were selected. We excluded patients with missing follow-up information, palliative treatments, chemotherapy, immunotherapy, ablative treatments, and radiation therapy other than SBRT or unknown information on the type of radiation therapy. Patients who underwent multimodal treatments or unspecified surgical procedures, liver transplantations, primarily bile duct resections with or without partial hepatectomy/liver transplantation, and missing treatment details were further excluded. Given that SBRT is frequently indicated in selective cohorts of patients who are not amenable to surgery owing to patients’ risk factors and poor performance status, we also excluded patients with contraindication to surgery due to “patient risk factors (comorbid conditions, advanced age, and so on)” in line with previous studies on comparisons between SBRT and surgery [21]. The histology ICD-O-3 codes 8170–8175 (hepatocellular carcinoma including fibrolamellar/scirrhous, spindle-cell variant, clear-cell, pleomorphic type, and not otherwise specified) were selected. Our final study population included 4153 patients.

The extracted clinicopathologic variables included age, sex, race, year of diagnosis, treating facility, the distance between residence and facility, educational attainment (high school degree), residence area, inferred annual household income, insurance status, comorbidities (Charlson/Deyo score), clinical stage, tumor size, TNM stage, and grading. The type of surgical procedure was obtained by the site-specific classification codes indicated by the procedure numbers 20 to 60, including wedge/segmental resection, lobectomy/extended lobectomy, hepatectomy not otherwise specified.

Biological effective dose (BED) was calculated as total dose × [1 + dose per fraction/(α/β)] [22]. To determine the total dose in Gray (Gy), the “number of fractions administered during treatment” and the “total dose of regional radiation therapy” measured in centigray (cGy) were multiplied and divided by 100. The dose per fractionation in Gray (Gy) was determined by the ratio of the “total dose of regional radiation therapy” measured in centigray (cGy) and the total number of fractions divided by 100. The radiosensitivity of the liver is estimated by α/β, with higher values having a lower sensitivity to the sparing effects of fractionations. In the current model, an α/β ratio of 10 was used, as recommended previously in the literature [23]. Given that dose-dependent effects of SBRT are discussed in the literature, we also stratified the SBRT cohort by the BED using 100 Gy as a cutoff value [24]. 

The primary endpoint was overall survival, measured from the time of cancer diagnosis to death of any cause or last follow-up. Secondary objectives were the effectiveness of SBRT on overall survival in patient subgroups (age, sex, comorbidities, facility type, tumor size, stage, and grade) and trends of SBRT utilization over time.

Propensity scores were calculated using a multivariate logistic regression model with the following covariates and type of treatment as the dependent variable: age, gender, race, era of diagnosis (2004–2009 vs. 2010–2015), facility type, the distance between residence and facility, income, insurance type, residence area, high school degree, comorbidity, tumor size, and clinical stage group. Variables collected after therapy initiation (e.g., pathologic stage) were not included in the model, in line with previous reports [25,26]. Patients who received SBRT were matched to patients who underwent surgery at a 1:2 ratio using a nearest neighbor matching algorithm (without replacement). A caliper width of 0.1 was used as the logit of the propensity score [27]. The success of propensity matching was assessed by comparing the standardized differences of the covariates between the groups. A standardized difference below 0.1 was considered as an indicator of balance.

Categorical data were assessed using the Pearson χ^2^ or Fisher’s exact test. Continuously scaled measures were analyzed using the Wilcoxon rank sum test or Student’s *t*-test. The primary outcome was calculated using the Kaplan–Meier method and compared using the stratified log-rank test for each treatment group as well as the subgroups’ clinical stage and BED. A multivariable Cox proportional hazards regression model adjusted for patient and facility factors was used to compare the association between oncologic outcomes as well as specific treatment details and overall survival. Correlation coefficients for the type of treatment and reported HCC cases were compared using the Fisher’s z transformation test. Subgroup analyses were performed within the matched study group for selective variables to explore the heterogeneity of treatment effects using tests of interaction, as described previously [26]. All statistical analyses and propensity matching were performed with R version 4.0.3 (Vienna, Austria). Graphpad was used for data visualization.

## 3. Results

### 3.1. Study Cohort Characteristics

Of 173,293 patients with HCC, we included 4153 patients, of whom 366 (9%) and 3787 (91%) received SBRT or surgery, respectively, as primary treatment for stage I/II HCC between 2004 and 2015 (Figure 1). Patients’ baseline characteristics are listed in Table 1. Most patients who underwent surgery received a segmentectomy (72%). In the unmatched dataset, patients who received SBRT were older (≥71: 38% vs. 27%; *p* < 0.001), more frequently of white ethnicity (93% vs. 82%; *p* < 0.001), and less likely to have any comorbidities (comorbidity index 0: 69% vs. 58%; *p* < 0.001). In the SBRT group, 20% and 17% lived in areas comprising the highest (≥29%) and lowest (<14%) high school graduation areas, while higher rates were observed in the surgery group, with 28% and 21%, respectively. Compared with patients who had surgery, fewer patients treated by SBRT had private insurance and lived in high-income areas (>63,333 USD), respectively. Receiving SBRT was also associated with treatment in non-academic facilities, higher tumor stage, and smaller tumor size (<3 cm). Matching by propensity score resulted in well-balanced groups for all covariates (Figure S1). 

### 3.2. SBRT Dose Selection and Clinical Practice Patterns

**Figure 2:**
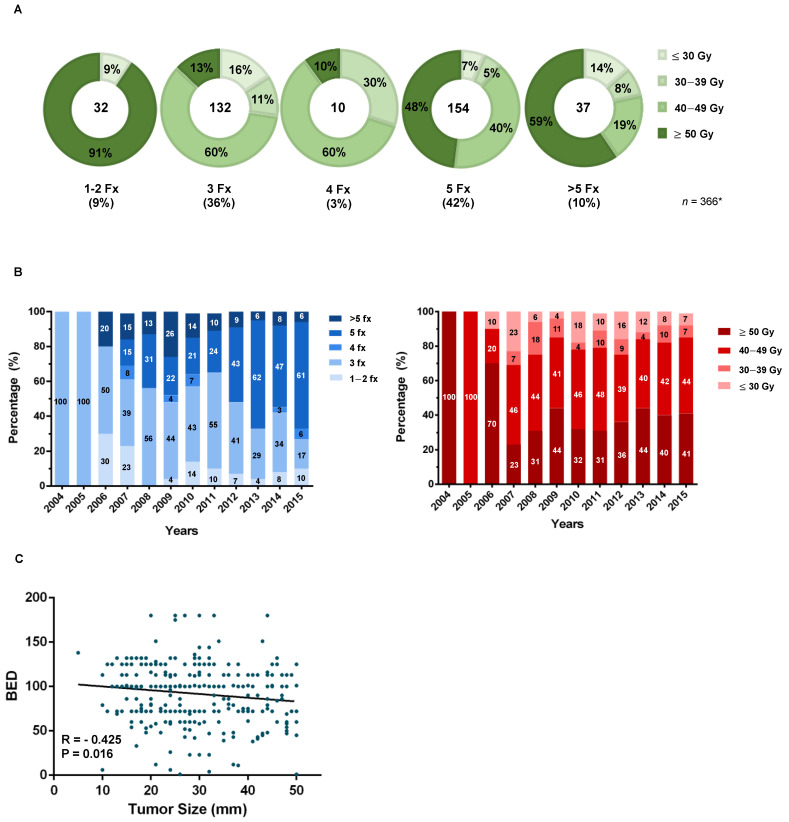
Characterization of SBRT treatment. Distribution of dose and fractionation regimens with total numbers and proportions are displayed for the study population with SBRT (*n* = 366) (**A**). Fx, fractionation regimen; * one patient with 50 Gy treatment had no data of the fractionation regimen. The use of dose and fractionation regimens over the study period is displayed (**B**). The relationship between BED and tumor size (**C**) is demonstrated in a scatter plot (Pearson’s correlation analysis).

In the SBRT cohort, most patients were treated with either five (42%) or three fractions (36%) (Figure 2A). While the radiation doses remained the same over time, with the majority of patients treated with dose regimens above 40 Gy (*p* = 0.844), the fractionation regimens have changed in favor of five fractions over time (*p* = 0.001) (Figure 2B). The most commonly applied fractionation doses were 40 Gy (14%) in five fractions and 50 Gy (14%) in five fractions. Other frequent dose fractionations included 48 Gy (10%) in three fractions and 45 Gy (11%) in three fractions. Therefore, the majority of patients received a dose per fraction between 40 and 49 Gy (42%) or ≥50 Gy (39%). These analyses of the SBRT regimens revealed significant heterogeneity across patients and institutions. The median BED was 100 Gy (interquartile range, 72 to 113 Gy). As expected, higher BED was observed in smaller tumor sizes (*p* = 0.016) (Figure 2C).

### 3.3. Survival Analysis

**Figure 3:**
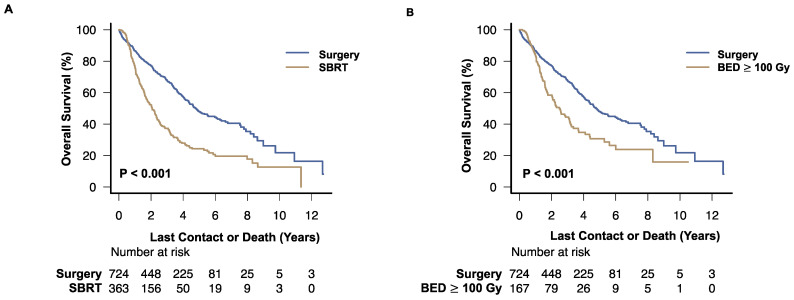
Overall survival in the propensity score matched study cohort with stage I or II hepatocellular carcinoma (log-rank test) (**A**). Overall survival in the subset of patients with a biologic equivalent dose (BED) ≥ 100 Gy versus surgery (**B**). SBRT, stereotactic body radiation therapy.

The median follow-up was 27 months (interquartile range, 13 to 48 months) in both groups after propensity score matching. During the study period, a total of 539 (50%) patients had died in the matched cohorts, with corresponding 5-year overall survival rates of 40% (95% CI 36–44%). Surgical resection was associated with a significant overall survival benefit in the unmatched cohort (unadjusted HR 0.43 (95% CI 0.37–0.49), *p* < 0.001), with a 1-year overall survival rate of 88% (95% CI 87–89%) versus 76% (95% CI 71–80%) and a 5-year overall survival rate of 52% (95% CI 50–53%) versus 24% (95% CI 19–30%) in the SBRT group (Figure 3A). In the propensity score cohort, the survival benefit of surgery was consistent (unadjusted HR 0.47 (95% CI 0.40–0.56); *p* < 0.001), with a 1-year overall survival rate of 87% (95% CI 84–89%) versus 76% (95% CI 71–80%) and a 5-year overall survival rate of 48% (95% CI 43–53%) versus 24% (95% CI 19–30%) in the SBRT group.

**Figure 4:**
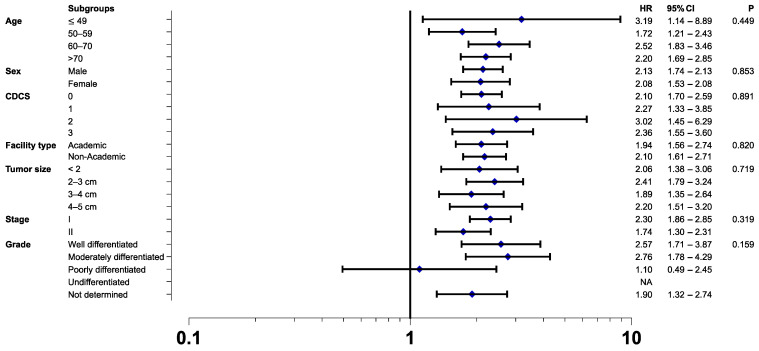
Forest plot depicting the hazard ratio of stereotactic body radiation therapy (SBRT) versus hepatectomy in the matched study cohort. Black squares represent hazard ratios (and 95% confidence intervals are represented by the corresponding horizontal lines).

A multivariable Cox regression analysis revealed the use of SBRT, advanced tumor stage, and tumor size as predictors of poor survival (Table 2). The favorable association of surgery with overall survival in the matched cohort was further reflected by subgroup analyses using tests of interaction of a priori defined subgroups. These analyses detected no heterogeneity of HR, as demonstrated by the forest plot in Figure 4. 

### 3.4. Effect of Treatment Details on Overall Survival

To explore the impact of treatment details on survival, we stratified the cohort for the different types of treatment in both groups. Patients who underwent segmentectomy, lobectomy, and hepatectomy (not further specified) had 5-year survival rates of 48% (95% CI 42–53%), 49% (95% CI 39–58%), and 58% (95% CI 33–76%), respectively (Figure S2A). The subset of SBRT patients with a BED ≥ 100 Gy demonstrated significantly higher 5-year overall survival rates (31%, 95% CI 22–40%) compared with patients treated with a BED < 100 Gy (14%, 95% CI 8–22%) (HR 0.61, 95% 0.47–0.81; *p* < 0.001) (Figure S2B). However, compared with patients in the surgery group, the overall survival difference between BED ≥ 100 Gy and surgical resection remained significant in favor of surgical resection (*p* < 0.001) (Figure 3B). Of note, the proportions of patients treated with BED ≥ 100 Gy were comparable between 2004–2009 and 2010–2015 (*n* = 120, 82% vs. *n* = 141, 84%, *p* = 0.649), while the survival benefit of surgical resection versus SBRT was higher in the era 2010–2015 (HR 0.41, 95% 0.33–0.50; *p* < 0.001) compared with the era 2004–2009 (HR 0.62, 95% 0.43–0.90; *p* = 0.01).

## 4. Discussion

Recently, SBRT has emerged as an effective treatment modality in localized HCC in patients who were no candidates for surgical resection. Two single-center retrospective studies (1:1 propensity score matching) compared SBRT versus surgical treatment and demonstrated similar 5-year survival rates of 74% versus 64% (SBRT: *n* = 33 vs. surgical resection: *n* = 33, *p* = 0.932) and 71% versus 71% (SBRT: *n* = 104 vs. surgical resection: *n* = 104; *p* = 0.673), respectively [13,15]. Another retrospective study including 81 patients with 1:2 propensity score matching revealed a lower 5-year survival rate after SBRT versus surgical resection of 48% versus 75%; *p* < 0.01 [14]. These studies were potentially affected by selection bias, resulting in large survival differences compared with other Western cohort studies using SBRT [26]. As large-scale data from prospective-controlled trials are currently not available in the literature, we conducted a propensity-score-matched analysis using the NCDB including 4153 patients to assess the effectiveness of SBRT compared with surgical resection as the current standard of care. Our results revealed that patients who underwent surgical resection as primary treatment experienced a significantly longer overall survival compared with those treated with SBRT, which was consistent across all subgroups assessed. Importantly, we detected no heterogeneity of treatment effects in clinically relevant subgroups. 

Several phase II trials have assessed the effectiveness and safety of SBRT, which extended the indication of SBRT to different stages of HCC [28,29,30,31]. Current evidence on comparisons of SBRT to other liver-directed treatment modalities is limited to retrospective studies and two meta-analyses of retrospective studies with conflicting results [32,33]. A multicentric retrospective study demonstrated that SBRT may have an advantage over ablation in terms of local tumor control at 3 years in “high-risk” locations (e.g., suphrenic, central, and adjacent to biliary structures) and in larger tumors (>3 cm), while having a comparable overall survival rate (radiofrequency ablation vs. SBRT: HR 0.85, 95% CI 0.67–1.07) [34]. In particular, tumors in subphrenic positions are prone to inadequate ablation by radiofrequency ablation owing to limited visibility and accessibility, which might result in higher local recurrence rates [35]. Another large cohort study using the NCDB revealed a survival benefit of radiofrequency ablation over SBRT (HR 0.67, 95% CI 0.55–0.81) in patients with stage I/II HCC [26]. The lack of high-level evidence and clinical uncertainty is reflected by inconsistent recommendations from different guidelines. Despite the lack of data from randomized trials comparing SBRT to locoregional treatments and surgical resection in the management of HCC, current AASLD and NCCN guidelines consider SBRT as an alternative treatment modality in patients with contraindication for ablation or resection, whereas the EASL and JSH do not because of the low level of evidence. 

Importantly, we discovered a wide variation in fractionation regimens and detected a shift from three to five fractions over the study period. About one-third of the included patients who received SBRT had 40–50 Gy in five fractions, which is a common ablative dose regimen [36,37]. However, dose regimens in SBRT are a matter of ongoing debate, which is mainly because of heterogeneous subgroups of patients with variable severity of underlying liver disease and tolerable liver volumes. A recent multicenter phase II trial including 74 patients with small, unresectable HCC (median tumor size 2.4 cm) demonstrated 45–60 Gy in three fractions to be associated with a low level of toxicity (2% CTCAE (common terminology criteria for adverse events) Grade 3) as well as high local tumor control and overall survival rates of 95% and 76% at 3 years [38]. In line with these data, another phase II trial on 90 patients with solitary HCC <4 cm treated with a dose regimen of 35–40 Gy in five fractions also revealed high local tumor control and overall survival rates of 96% and 67% at 3 years with low toxicity levels of 2% (CTCAE Grade 3) [39]. Others suggested individualized dosimetric plans based on indocyanine green retention to achieve local control rates of 95% and high safety in advanced cirrhotic livers [40]. However, the minimum dose required for tumor control remains unclear and a matter of ongoing study (NCT02460835). A recent large-scale multicenter retrospective trial including 602 patients with unresectable HCC used dose regimens ranging from 28 to 55 Gy in 1–6 fractions and indicated that higher doses with BED ≥ 100 Gy are associated with a survival benefit [41]. Our study confirmed an association with prolonged overall survival for patients treated with a BED ≥ 100 Gy compared with the subset of patients treated with BED < 100 Gy. Previous reports showed that a higher BED was applied in smaller tumors compared with larger tumors (>4 cm) due to the volumetric dose constraints of the liver [42]. We confirmed this finding of a higher BED in smaller tumors, although we applied strict selection criteria with tumors < 5 cm as opposed to other reports of the NCDB, including large lesions up to 17 cm [23]. Our analyses showed no further benefit of SBRT compared with different types of surgical resections, as the use of SBRT has been suggested to be an alternative to extended resections, as shown in other cancer entities (e.g., non-small-cell lung cancer) [43,44]. Nevertheless, there are no clinical practice guidelines of the American Society for Radiation Oncology for the SBRT treatment of HCC and a consensus on BED doses is lacking.

There are several limitations of our study. As a retrospective analysis of a large database including several U.S. institutions, our results might be affected by inter-institutional heterogeneity with respect to the patients included and the treatments applied [45]. During the study period, significant refinements of the SBRT technique have been made globally, including technological advances in radiation therapy delivery systems (e.g., volumetric modulation arc therapy), the use of image-guided radiation therapy (e.g., CT, MRI, and PET scans), and motion management techniques, which resulted in better outcomes [46]. Yet, we did not observe a survival benefit of patients treated with SBRT between 2010–2015 and 2004–2009. This might be due to heterogenous application of SBRT techniques between institutions, including the shift to hypofractionation from three to five fractions over time, as well as the use of heterogenous SBRT doses, and the results should be considered therefore cautiously. Moreover, the lack of data on known prognostic variables such as patients’ liver function treatment-associated toxicity and postoperative liver surgery complications [47,48,49], as well as the number and location of lesions (i.e., centrally located tumors with potential vascular invasion), and other second-line treatments after primary surgery or SBRT needs to be mentioned as a limitation of our study that might have introduced bias. Further, there are no data on the treatment of recurrent disease or data on local tumor control. Hence, our results could overestimate or underestimate the treatment effects in both groups. To reduce bias, we limited our analyses to stage I/II HCC using a rigorous statistical approach including propensity score matching. Despite these actions and the exclusion of patients with contraindications to surgery because of their poor performance status, we cannot fully preclude possible selection bias in favor of the surgical resection group. However, the superiority of surgical resection was confirmed across all subgroups and in the multi-variable analysis. In addition, we detected that a BED ≥ 100 Gy is associated with a survival benefit, although we could not further evaluate the reasons for BED differences on individual patient-level data. Using this subgroup of SBRT, surgical resection was still associated with a significantly longer overall survival, which further supports its long-term treatment benefit.

## 5. Conclusions

In conclusion, our findings suggest that, in patients with stage I/II HCC, surgical resection might be superior to SBRT in terms of overall survival. The lack of inter-institutional consensus with respect to SBRT fractionation and dose regimens is a further important finding of our study. These findings should prompt confirmatory randomized trials with standardized dose regimens to define the role of SBRT in the management of localized HCC. Until the results of these data are available, the use of SBRT should be limited to clinical trials and patients who are no surgical candidates [50].

## Figures and Tables

**Table 1:** Demographic and clinical characteristics of patients with hepatocellular carcinoma.

	Unmatched Dataset			Matched Dataset		
	SBRT	Surgery		SBRT	Surgery	
Characteristics	*n* = 366	*n* = 3787	*p*	*n* = 363	*n* = 724	*p*
**Age, years**			<0.001			0.960
≤49	10 (3)	277 (7)		10 (3)	21 (3)	
50–59	92 (25)	1035 (27)		91 (25)	173 (24)	
60–70	126 (34)	1458 (39)		125 (34)	246 (34)	
≥71	138 (38)	1017 (27)		137 (38)	284 (39)	
**Sex**			0.077			0.945
Male	249 (68)	2742 (72)		248 (68)	492 (68)	
Female	117 (32)	1045 (28)		115 (32)	232 (32)	
**Race**			<0.001			0.478
White	340 (93)	3099 (82)		337 (93)	685 (95)	
Black	20 (6)	647 (17)		20 (5)	31 (4)	
Other/Unknown	6 (1)	41 (1)		6 (2)	8 (1)	
**Comorbidity Index**			<0.001			0.970
0	253 (69)	2179 (58)		251 (69)	489 (67)	
1	40 (11)	990 (26)		40 (11)	84 (12)	
2	20 (6)	305 (8)		20 (6)	43 (6)	
3	53 (14)	313 (8)		52 (14)	108 (15)	
**Era of Diagnosis**			<0.001			0.398
2004–2009	69 (19)	1081 (28)		69 (19)	122 (17)	
2010–2015	297 (81)	2706 (72)		294 (81)	602 (83)	
**Facility Type**			0.007			0.967
Community	8 (2)	83 (2)		8 (2)	13 (2)	
Comprehensive Community	66 (18)	702 (18)		66 (18)	133 (18)	
Academic/research	235 (64)	2523 (67)		233 (64)	467 (65)	
Integrated network	57 (16)	418 (11)		56 (16)	111 (15)	
Missing	0	61 (2)		0	0	
**Distance to Facility**			0.082			0.902
<12.5 miles	166 (45)	1832 (48)		166 (46)	342 (47)	
12.5–50 miles	114 (31)	1146 (30)		113 (31)	213 (29)	
50–250 miles	83 (22)	723 (19)		81 (22)	164 (23)	
>250 miles	2 (1)	79 (2)		2 (1)	2 (1)	
Missing	1 (1)	7 (1)		1 (1)	3 (1)	
**High School Education**			<0.001			0.939
≥29%	72 (20)	1043 (28)		72 (20)	155 (21)	
20–29%	102 (28)	918 (24)		102 (28)	188 (26)	
14–20%	125 (34)	987 (26)		122 (34)	250 (35)	
<14%	62 (17)	790 (21)		62 (17)	121 (17)	
Missing	5 (1)	49 (1)		5 (1)	10 (1)	
**Residence Area**			0.135			0.997
Metro	305 (83)	3263 (86)		303 (84)	601 (83)	
Urban	49 (13)	371 (10)		48 (13)	96 (13)	
Rural	2 (1)	47 (1)		2 (1)	5 (1)	
Missing	10 (3)	106 (3)		10 (3)	22 (3)	
**Insurance**			<0.001			0.991
Not insured	9 (3)	122 (3)		9 (3)	22 (3)	
Private	91 (25)	1378 (36)		91 (25)	178 (25)	
Medicaid	39 (11)	452 (12)		39 (11)	78 (11)	
Medicare	220 (60)	1725 (46)		217 (60)	431 (60)	
Other	6 (2)	59 (2)		6 (2)	14 (2)	
Unknown	1 (1)	51 (1)		1 (1)	1 (1)	
**Median Household Income, USD**			0.001			0.997
<40,227	77 (21)	813 (22)		77 (21)	151 (21)	
40,227–50,353	105 (29)	791 (21)		104 (29)	214 (30)	
50,354–63,332	91 (25)	890 (24)		90 (25)	180 (25)	
>63,333	87 (24)	1243 (33)		87 (24)	168 (23)	
Missing	6 (1)	50 (1)		5 (1)	11 (1)	
**Tumor Stage**			0.018			0.230
Stage I	250 (68)	2809 (74)		248 (68)	521 (72)	
Stage II	116 (32)	978 (26)		115 (32)	203 (28)	
**Tumor Size, cm**			0.002			0.666
<2	93 (25)	769 (20)		91 (25)	173 (24)	
2–3	129 (35)	1136 (30)		128 (35)	236 (33)	
3–4	81 (22)	1032 (27)		81 (22)	182 (25)	
4–5	63 (17)	850 (22)		63 (17)	133 (18)	
**Surgery**			-			-
Segmentectomy		2734 (72)			535 (74)	
Lobectomy		896 (24)			162 (22)	
Hepatectomy (NOS)		157 (4)			27 (4)	

Data are given as no. (%) unless otherwise noted. Pearson χ^2^ or Fisher’s exact test was performed between the study groups dependent on sample size. Percentages were rounded and may not total 100. Abbreviations: USD, U.S. dollar; NOS, not otherwise specified.

**Table 2:** Predictors of death (overall survival) of patients with hepatocellular carcinoma in the matched dataset.

	Adjusted HR ^a^	95% CI	
Variable			*p*
**Treatment**			
SBRT	Reference		
Surgery	0.44	0.37–0.53	<0.001
**Tumor Stage**			
Stage I	Reference		
Stage II	1.76	1.47–2.11	<0.001
**Tumor Size, cm**			
<2	Reference		
2–3	1.40	1.09–1.79	0.008
3–4	1.45	1.11–1.88	0.006
4–5	1.65	1.25–2.18	0.001
**Grading**			
Well differentiated	Reference		
Moderately differentiated	0.84	0.66–1.09	0.191
Poorly differentiated	1.53	1.14–2.12	0.005
Undifferentiated	1.16	0.36–3.72	0.796
Not determined	1.83	1.43–2.34	<0.001
**BED ^b^**			
<100 Gray	Reference		
≥100 Gray	0.58	0.43–0.77	<0.001

^a^ Adjustments of patient and facility factors were accounted for in this model (age, sex, race, facility type, facility distance, education, insurance status, comorbidity index, year of diagnosis, area of living, and household income). ^b^ Only in the subset of patients treated with SBRT. Abbreviations: HR, hazard ratio; BED, biological effective dose.

## Data Availability

The data underlying this article were provided by the National Cancer Database by permission. The National Cancer Data Base (NCDB) is a joint project of the Commission on Cancer (CoC) of the American College of Surgeons and the American Cancer Society. The CoC’s NCDB and the hospitals participating in the CoC NCDB are the source of the de-identified data used herein; they have not verified and are not responsible for the statistical validity of the data analysis or the conclusions derived by the authors.

## Supplementary Materials

The following supporting information can be downloaded at https://www.mdpi.com/article/10.3390/cancers15082330/s1. Figure S1: Covariate balance measured by the standardized mean difference in the unmatched and matched cohort; Figure S2: Overall survival stratified by (A) the type of surgical treatment and (B) biologic equivalent dose (BED) ≥ 100 Gy and BED < 100 Gy in the propensity-score-matched study cohort (log-rank test).

## References

[1] Sung H., Ferlay J., Siegel R.L., Laversanne M., Soerjomataram I., Jemal A., Bray F. (2021). Global Cancer Statistics 2020: Globocan Estimates of Incidence and Mortality Worldwide for 36 Cancers in 185 Countries. CA Cancer J. Clin..

[2] Lin L., Yan L., Liu Y., Qu C., Ni J., Li H. (2020). The Burden and Trends of Primary Liver Cancer Caused by Specific Etiologies from 1990 to 2017 at the Global, Regional, National, Age, and Sex Level Results from the Global Burden of Disease Study 2017. Liver Cancer.

[3] Rahbari N.N., Mehrabi A., Mollberg N.M., Muller S.A., Koch M., Buchler M.W., Weitz J. (2011). Hepatocellular Carcinoma: Current Management and Perspectives for the Future. Ann. Surg..

[4] Marrero J.A., Kulik L.M., Sirlin C.B., Zhu A.X., Finn R.S., Abecassis M.M., Roberts L.R., Heimbach J.K. (2018). Diagnosis, Staging, and Management of Hepatocellular Carcinoma: 2018 Practice Guidance by the American Association for the Study of Liver Diseases. Hepatology.

[5] European Association for the Study of the Liver (2018). Electronic address, easloffice easloffice eu, and Liver European Association for the Study of the. “Easl Clinical Practice Guidelines: Management of Hepatocellular Carcinoma”. J. Hepatol..

[6] Benson A.B., D’Angelica M.I., Abbott D.E., Abrams T.A., Alberts S.R., Anaya D.A., Anders R., Are C., Brown D., Chang D.T. (2019). Nccn Guidelines Insights: Hepatobiliary Cancers, Version 2.2019: Featured Updates to the Nccn Guidelines. J. Natl. Compr. Cancer Netw..

[7] Kudo M., Kawamura Y., Hasegawa K., Tateishi R., Kariyama K., Shiina S., Toyoda H., Imai Y., Hiraoka A., Ikeda M. (2021). Management of Hepatocellular Carcinoma in Japan: JSH Consensus Statements and Recommendations 2021 Update. Liver Cancer.

[8] Robertson J.M., Lawrence T.S., Dworzanin L.M., Andrews J.C., Walker S., Kessler M.L., DuRoss D.J., Ensminger W.D. (1993). Treatment of Primary Hepatobiliary Cancers with Conformal Radiation Therapy and Regional Chemotherapy. J. Clin. Oncol..

[9] Birgin E., Rasbach E., Seyfried S., Rathmann N., Diehl S.J., Schoenberg S.O., Reissfelder C., Rahbari N.N. (2020). Contralateral Liver Hypertrophy and Oncological Outcome Following Radioembolization with (90)Y-Microspheres: A Systematic Review. Cancers.

[10] Murray L., Dawson L.A. (2017). Advances in Stereotactic Body Radiation Therapy for Hepatocellular Carcinoma. Semin. Radiat. Oncol..

[11] Mathew A.S., Atenafu E.G., Owen D., Maurino C., Brade A., Brierley J., Dinniwell R., Kim J., Cho C., Ringash J. (2020). Long Term Outcomes of Stereotactic Body Radiation Therapy for Hepatocellular Carcinoma without Macrovascular Invasion. Eur. J. Cancer.

[12] Long Y., Liang Y., Li S., Guo J., Wang Y., Luo Y., Wu Y. (2021). Therapeutic Outcome and Related Predictors of Stereotactic Body Radiotherapy for Small Liver-Confined Hcc: A Systematic Review and Meta-Analysis of Observational Studies. Radiat. Oncol..

[13] Sun J., Wang Q., Hong Z.X., Li W.G., He W.P., Zhang T., Zhang A.M., Fan Y.Z., Sun Y.Z., Zheng L. (2020). Stereotactic Body Radiotherapy Versus Hepatic Resection for Hepatocellular Carcinoma (</= 5 cm): A Propensity Score Analysis. Hepatol. Int..

[14] Nakano R., Ohira M., Kobayashi T., Ide K., Tahara H., Kuroda S., Shimizu S., Kimura T., Nagata Y., Aikata H. (2018). Hepatectomy Versus Stereotactic Body Radiotherapy for Primary Early Hepatocellular Carcinoma: A Propensity-Matched Analysis in a Single Institution. Surgery.

[15] Su T.-S., Liang P., Liang J., Lu H.-Z., Jiang H.-Y., Cheng T., Huang Y., Tang Y., Deng X. (2017). Long-Term Survival Analysis of Stereotactic Ablative Radiotherapy Versus Liver Resection for Small Hepatocellular Carcinoma. Int. J. Radiat. Oncol. Biol. Phys..

[16] Xu X.-L., Liu X.-D., Liang M., Luo B.-M. (2018). Radiofrequency Ablation versus Hepatic Resection for Small Hepatocellular Carcinoma: Systematic Review of Randomized Controlled Trials with Meta-Analysis and Trial Sequential Analysis. Radiology.

[17] Xu J., Zhao Y. (2015). Comparison of Percutaneous Microwave Ablation and Laparoscopic Resection in the Prognosis of Liver Cancer. Int. J. Clin. Exp. Pathol..

[18] Reig M., Forner A., Rimola J., Ferrer-Fàbrega J., Burrel M., Garcia-Criado Á., Kelley R.K., Galle P.R., Mazzaferro V., Salem R. (2021). Bclc Strategy for Prognosis Prediction and Treatment Recommendation: The 2022 Update. J. Hepatol..

[19] Winchester D.P., Stewart A.K., Phillips J.L., Ward E.E. (2010). The National Cancer Data Base: Past, Present, and Future. Ann. Surg. Oncol..

[20] von Elm E., Altman D.G., Egger M., Pocock S.J., Gotzsche P.C., Vandenbroucke J.P., STROBE Initiative (2007). The Strengthening the Reporting of Observational Studies in Epidemiology (STROBE) Statement: Guidelines for Reporting Observational Studies. PLoS Med..

[21] Mayne N.R., Lin B.K., Darling A.J., Raman V., Patel D.C., Liou D.Z., D’amico T.A., Yang C.-F.J. (2020). Stereotactic Body Radiotherapy Versus Delayed Surgery for Early-Stage Non-Small-Cell Lung Cancer. Ann. Surg..

[22] Fowler J.F. (2010). 21 years of Biologically Effective Dose. Br. J. Radiol..

[23] Robbins J.R., Schmid R.K., Hammad A.Y., Gamblin T.C., Erickson B.A. (2019). Stereotactic Body Radiation Therapy for Hepatocellular Carcinoma: Practice Patterns, Dose Selection and Factors Impacting Survival. Cancer Med..

[24] Schaub S., Hartvigson P.E., Lock M., Høyer M., Brunner T.B., Cardenes H.R., Dawson L., Kim E.Y., Mayr N.A., Lo S.S. (2018). Stereotactic Body Radiation Therapy for Hepatocellular Carcinoma: Current Trends and Controversies. Technol. Cancer Res. Treat..

[25] Mokdad A.A., Minter R.M., Zhu H., Augustine M.M., Porembka M., Wang S., Yopp A.C., Mansour J.C., Choti M.A., Polanco P.M. (2017). Neoadjuvant Therapy Followed by Resection Versus Upfront Resection for Resectable Pancreatic Cancer: A Propensity Score Matched Analysis. J. Clin. Oncol..

[26] Rajyaguru D.J., Borgert A.J., Smith A.L., Thomes R.M., Conway P.D., Halfdanarson T., Truty M.J., Kurup A.N., Go R.S. (2018). Radiofrequency Ablation Versus Stereotactic Body Radiotherapy for Localized Hepatocellular Carcinoma in Nonsurgically Managed Patients: Analysis of the National Cancer Database. J. Clin. Oncol..

[27] Austin P.C. (2013). The Use of Propensity Score Methods with Survival or Time-to-Event Outcomes: Reporting Measures of Effect Similar to Those Used in Randomized Experiments. Stat. Med..

[28] Yoon S.M., Kim S.Y., Lim Y.S., Kim K.M., Shim J.H., Lee D., An J., Jung J., Kim J.H., Lee H.C. (2020). Stereotactic Body Radiation Therapy for Small (≤5 cm) Hepatocellular Carcinoma Not Amenable to Curative Treatment: Results of a Single-Arm, Phase Ii Clinical Trial. Clin. Mol. Hepatol..

[29] Beaton L., Dunne E., Yeung R., Rackley T., Weber B., Mar C., Yong-Hing C., Yoshida E., DeVries K., Lee R. (2020). Stereotactic Body Radiotherapy for Large Unresectable Hepatocellular Carcinomas—A Single Institution Phase II Study. Clin. Oncol. (R. Coll. Radiol.).

[30] Durand-Labrunie J., Baumann A.-S., Ayav A., Laurent V., Boleslawski E., Cattan S., Bogart E., Le Deley M.-C., Steen V., Lacornerie T. (2020). Curative Irradiation Treatment of Hepatocellular Carcinoma: A Multicenter Phase 2 Trial. Int. J. Radiat. Oncol. Biol. Phys..

[31] Kimura T., Takeda A., Sanuki N., Ariyoshi K., Yamaguchi T., Imagumbai T., Katoh N., Eriguchi T., Oku Y., Ozawa S. (2020). Multicenter Prospective Study of Stereotactic Body Radiotherapy for Previously Untreated Solitary Primary Hepatocellular Carcinoma: The Strsph Study. Hepatol. Res..

[32] Lee J., Shin I.-S., Yoon W.S., Koom W.S., Rim C.H. (2020). Comparisons between Radiofrequency Ablation and Stereotactic Body Radiotherapy for Liver Malignancies: Meta-Analyses and a Systematic Review. Radiother. Oncol..

[33] Pan Y.-X., Fu Y.-Z., Hu D.-D., Long Q., Wang J.-C., Xi M., Liu S.-L., Xu L., Liu M.-Z., Chen M.-S. (2020). Stereotactic Body Radiotherapy vs. Radiofrequency Ablation in the Treatment of Hepatocellular Carcinoma: A Meta-Analysis. Front. Oncol..

[34] Kim N., Cheng J., Jung I., Liang J., Shih Y.L., Huang W.Y., Kimura T., Lee V.H.F., Zeng Z.C., Zhenggan R. (2020). Stereotactic Body Radiation Therapy Vs. Radiofrequency Ablation in Asian Patients with Hepatocellular Carcinoma. J. Hepatol..

[35] Kim N., Kim H.J., Won J.Y., Kim D.Y., Han K.-H., Jung I., Seong J. (2019). Retrospective Analysis of Stereotactic Body Radiation Therapy Efficacy over Radiofrequency Ablation for Hepatocellular Carcinoma. Radiother. Oncol..

[36] Sanuki N., Takeda A., Oku Y., Mizuno T., Aoki Y., Eriguchi T., Iwabuchi S., Kunieda E. (2013). Stereotactic Body Radiotherapy for Small Hepatocellular Carcinoma: A Retrospective Outcome Analysis in 185 Patients. Acta Oncol..

[37] Wang P.-M., Hsu W.-C., Chung N.-N., Chang F.-L., Jang C.-J., Fogliata A., Scorsetti M., Cozzi L. (2014). Feasibility of Stereotactic Body Radiation Therapy with Volumetric Modulated Arc Therapy and High Intensity Photon Beams for Hepatocellular Carcinoma Patients. Radiat. Oncol..

[38] Jang W.I., Bae S.H., Kim M., Han C.J., Park S.C., Kim S.B., Cho E., Choi C.W., Kim K.S., Hwang S. (2019). A Phase 2 Multicenter Study of Stereotactic Body Radiotherapy for Hepatocellular Carcinoma: Safety and Efficacy. Cancer.

[39] Takeda A., Sanuki N., Tsurugai Y., Iwabuchi S., Matsunaga K., Ebinuma H., Imajo K., Aoki Y., Saito H., Kunieda E. (2016). Phase 2 Study of Stereotactic Body Radiotherapy and Optional Transarterial Chemoembolization for Solitary Hepatocellular Carcinoma Not Amenable to Resection and Radiofrequency Ablation. Cancer.

[40] Feng M., Suresh K., Schipper M.J., Bazzi L., Ben-Josef E., Matuszak M.M., Parikh N.D., Welling T.H., Normolle D., Haken R.K.T. (2018). Individualized Adaptive Stereotactic Body Radiotherapy for Liver Tumors in Patients at High Risk for Liver Damage: A Phase 2 Clinical Trial. JAMA Oncol..

[41] Su T.-S., Liu Q.-H., Zhu X.-F., Liang P., Liang S.-X., Lai L., Zhou Y., Huang Y., Cheng T., Li L.-Q. (2021). Optimal Stereotactic Body Radiotherapy Dosage for Hepatocellular Carcinoma: A Multicenter Study. Radiat. Oncol..

[42] Kuo H.-T., Que J., Lin L.-C., Yang C.-C., Koay L.-B., Lin C.-H. (2017). Impact of Tumor Size on Outcome after Stereotactic Body Radiation Therapy for Inoperable Hepatocellular Carcinoma. Medicine.

[43] Chi A., Fang W., Sun Y., Wen S. (2019). Comparison of Long-Term Survival of Patients with Early-Stage Non-Small Cell Lung Cancer after Surgery Vs Stereotactic Body Radiotherapy. JAMA Netw. Open.

[44] Shirvani S.M., Jiang J., Chang J.Y., Welsh J., Likhacheva A., Buchholz T.A., Swisher S.G., Smith B.D. (2014). Lobectomy, Sublobar Resection, and Stereotactic Ablative Radiotherapy for Early-Stage Non-Small Cell Lung Cancers in the Elderly. JAMA Surg..

[45] Birgin E., Kaslow S.R., Hetjens S., Correa-Gallego C., Rahbari N.N. (2021). Minimally Invasive versus Open Liver Resection for Stage I/Ii Hepatocellular Carcinoma. Cancers.

[46] Méndez Romero A., de Man R.A. (2016). Stereotactic Body Radiation Therapy for Primary and Metastatic Liver Tumors: From Technological Evolution to Improved Patient Care. Best Pract. Res. Clin. Gastroenterol..

[47] Birgin E., Mehrabi A., Sturm D., Reißfelder C., Weitz J., Rahbari N.N. (2021). Infrahepatic Inferior Vena Cava Clamping Does Not Increase the Risk of Pulmonary Embolism Following Hepatic Resection. World J. Surg..

[48] Birgin E., Tesfazgi W., Knoth M., Wilhelm T., Post S., Rückert F. (2018). Evaluation of the New Isgls Definitions of Typical Posthepatectomy Complications. Scand. J. Surg..

[49] Rahbari N.N., Birgin E., Sturm D., Schwanebeck U., Weitz J., Reissfelder C. (2020). Randomized Clinical Trial of Biofoam(R) Surgical Matrix to Achieve Hemostasis after Liver Resection. HPB (Oxford).

[50] Meyer T. (2020). Stereotactic Body Radiotherapy for Hepatocellular Carcinoma—Still Searching for a Role. J. Hepatol..

