# Supplementary material for: Stereotactic Body Radiation Therapy versus Surgical Resection for Stage I/II Hepatocellular Carcinoma
Source: Cancers (Basel). 2023 Apr 17;15(8):2330. doi: 10.3390/cancers15082330 (PMC10136632; doi:10.3390/cancers15082330)
Supplement: Supplementary file 1 [file cancers-15-02330-s001.zip › cancers-2254104-supplementary.pdf]

Supplemental Data

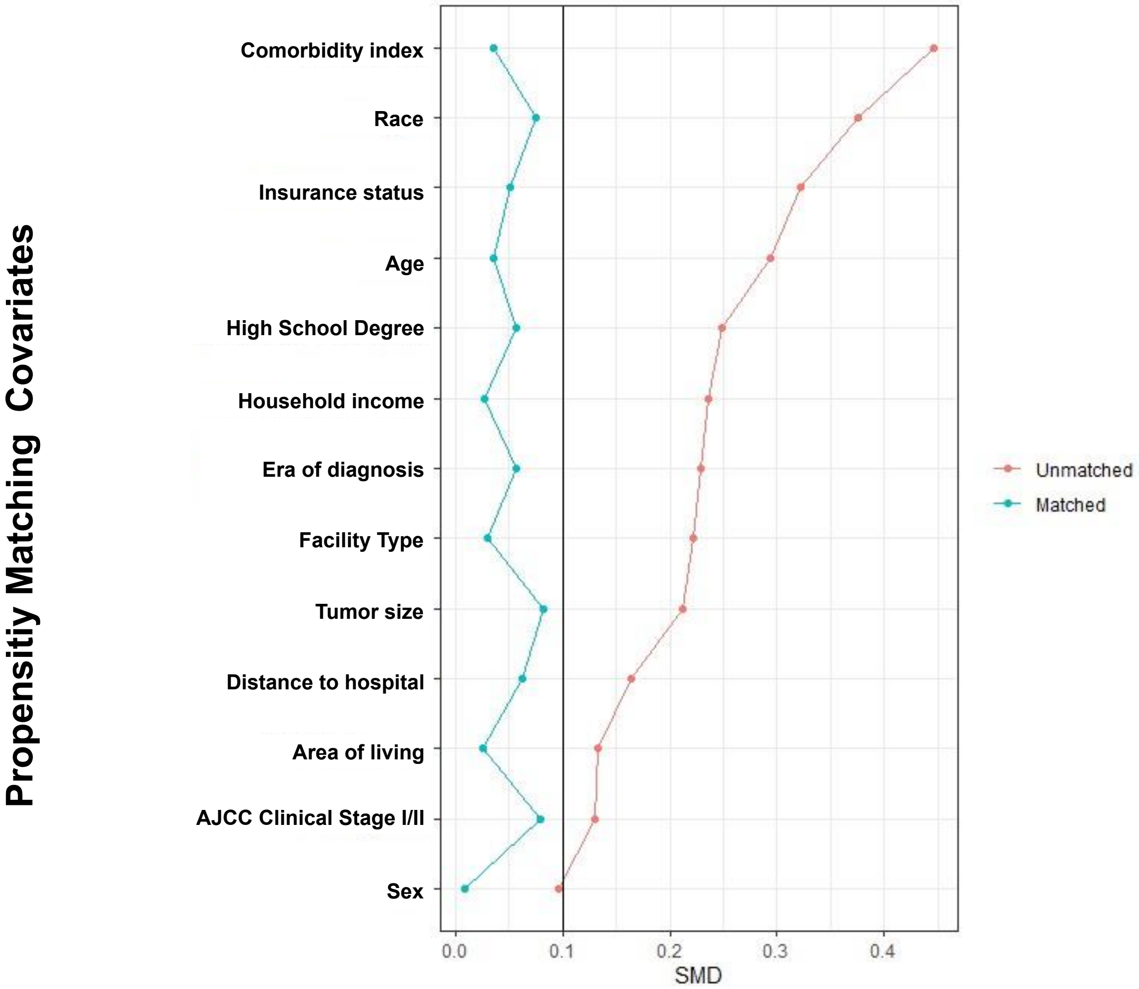

Figure S1: Covariate balance measured by the standardized mean difference in the unmatched and matched cohort

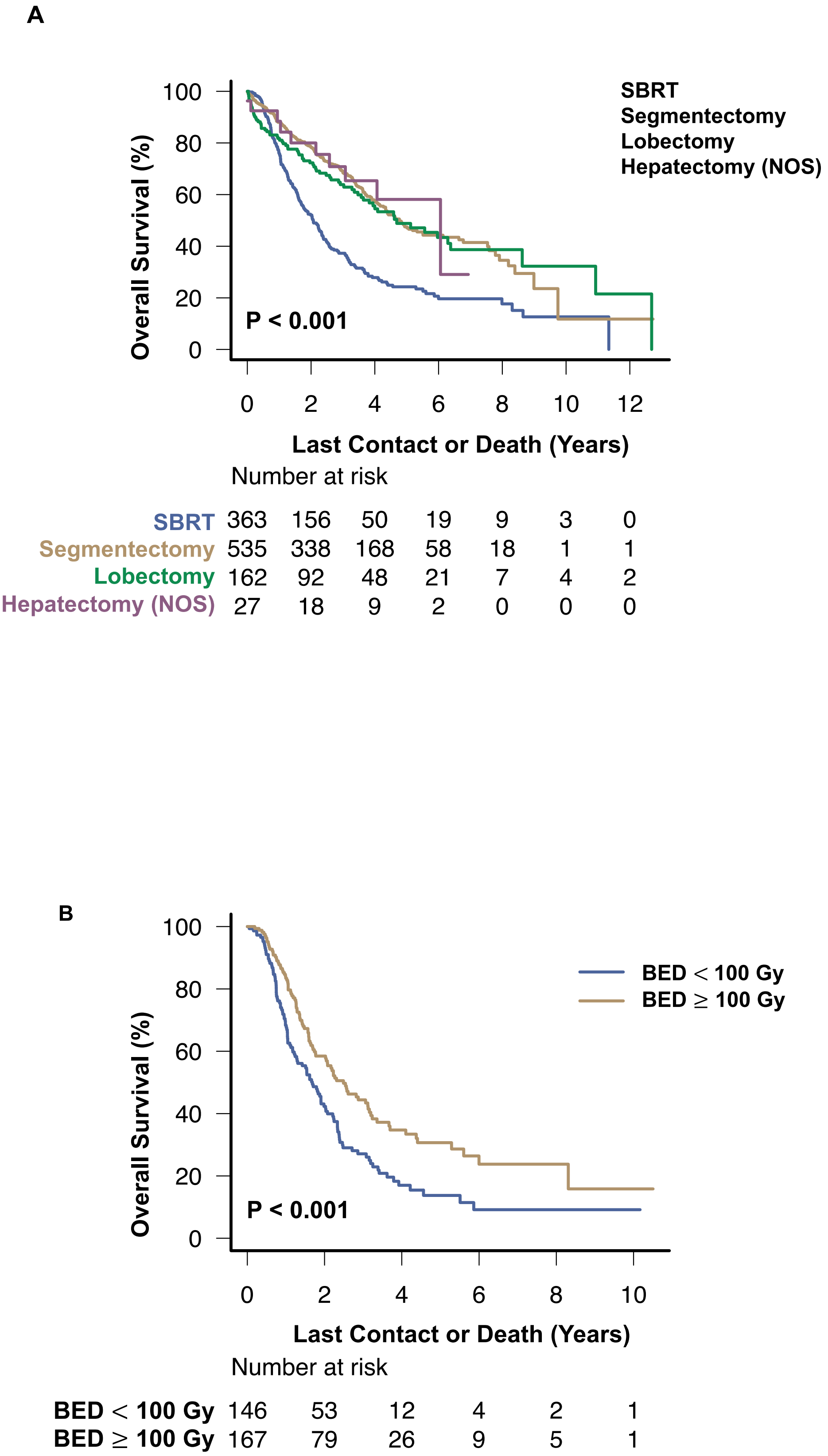

Figure S2: Overall survival stratified by (A) the type of surgical treatment and (B) biologic equivalent dose (BED) ≥ 100 Gy and BED < 100 Gy in the propensity score matched study cohort (log-rank test). NOS, not otherwise specified
